# The COVID-19 pandemic and Bitcoin: Perspective from investor attention

**DOI:** 10.3389/fpubh.2023.1147838

**Published:** 2023-04-12

**Authors:** Jieru Wan, You Wu, Panpan Zhu

**Affiliations:** ^1^Nottingham University Business School, University of Nottingham, Nottingham, United Kingdom; ^2^School of Economics, Beijing Technology and Business University, Beijing, China

**Keywords:** investor attention, Bitcoin, COVID-19 pandemic, VAR analysis, forecast

## Abstract

The response of the Bitcoin market to the novel coronavirus (COVID-19) pandemic is an example of how a global public health crisis can cause drastic market adjustments or even a market crash. Investor attention on the COVID-19 pandemic is likely to play an important role in this response. Focusing on the Bitcoin futures market, this paper aims to investigate whether pandemic attention can explain and forecast the returns and volatility of Bitcoin futures. Using the daily Google search volume index for the “coronavirus” keyword from January 2020 to February 2022 to represent pandemic attention, this paper implements the Granger causality test, Vector Autoregression (VAR) analysis, and several linear effects analyses. The findings suggest that pandemic attention is a granger cause of Bitcoin returns and volatility. It appears that an increase in pandemic attention results in lower returns and excessive volatility in the Bitcoin futures market, even after taking into account the interactive effects and the influence of controlling other financial markets. In addition, this paper carries out the out-of-sample forecasts and finds that the predictive models with pandemic attention do improve the out-of-sample forecast performance, which is enhanced in the prediction of Bitcoin returns while diminished in the prediction of Bitcoin volatility as the forecast horizon is extended. Finally, the predictive models including pandemic attention can generate significant economic benefits by constructing portfolios among Bitcoin futures and risk-free assets. All the results demonstrate that pandemic attention plays an important and non-negligible role in the Bitcoin futures market. This paper can provide enlightens for subsequent research on Bitcoin based on investor attention sparked by public emergencies.

## 1. Introduction and literature review

### 1.1. Research background and motivation

Since the World Health Organization (WHO) declared the novel coronavirus (COVID-19) pandemic a public health emergency of international concern on January 31, 2020, there have been more than 650 million confirmed cases of COVID-19 globally as of December 23, 2022, including a staggering 6.6 million fatalities.^1^ Almost all countries have been severely impacted by the pandemic (1). Particularly, Europe and the Americas have been the most affected regions, accounting for 41 and 28%, respectively, of the global confirmed cases. Unfortunately, these staggering numbers may also understate the damage of the pandemic, especially in developing countries (2). The pandemic has become the most significant public health event since the outbreak of the 1918 flu, with substantial implications for human health, society, and global economic growth (3). In order to address the threats posed by the pandemic, countries have implemented effective prevention and control measures, such as non-pharmaceutical interventions and vaccination (4). Nevertheless, COVID-19 has a high propensity to mutate, leading to the creation of many variant strains. Currently, the Omicron variant of COVID-19 and its multiple sublineages are rapidly spreading around the world due to their highly contagious nature (5, 6).

Due to the uncertainty of loss and the complexity of governance, along with extensive media coverage, the pandemic has had a powerful effect on investor psychology and expectations (7, 8). This further changes the behavior of investors, prompting them to adjust the allocation of investment portfolios in response to the high degree of uncertainty from the pandemic (7–9). Moreover, investors are now more likely to acquire news through the internet to make their investing decisions, which may speed up the digestion of emergency information such as the pandemic by the financial market (10, 11), leading to price responses and increased volatility (12). Specifically, increased investor attention to new information, like the pandemic, can generate more informational discoveries, resulting in temporary price pressure and reducing return predictability (13). Meanwhile, more investor attention may generate a lot of noise, and further cause significant volatility *via* the herd effect among investors (14). Numerous studies back up this perspective by using internet information to measure investor attention on the pandemic (i.e., pandemic attention) (10, 11, 15–17).

Bitcoin, as an essential asset for portfolio diversification, has attracted increasing attention from investors all over the world due to its volatility persistence, possible hedging properties (18, 19), and potential safe-haven capabilities (20–22). During the COVID-19 pandemic, lockdowns and social distancing measures have disrupted traditional in-person goods and services (23), driving investors to demand more digital goods and digital finance (24–26). And more importantly, the large-scale economic stimulus measures during the pandemic have caused fiscal deficits, currency devaluation, and political instability (27), prompting more and more investors to incorporate cryptocurrencies like Bitcoin into their portfolios in order to guard against uncertainties of traditional regularized-systems and protect their purchasing power (23, 27). However, the price of Bitcoin fell for a long time during the pandemic (28). And there is a drastic increase in volatility in all financial markets during the pandemic (29). In reality, returns and volatility play essential roles in asset pricing, risk management and portfolio allocation (30). It is therefore natural to discuss whether Bitcoin returns and volatility are affected by pandemic attention. Numerous academic studies have examined the determinants of Bitcoin returns and volatility (18, 31–34). However, there is little literature focusing on investor attention. Essentially, investor attention is perceived as an awareness of whether or not information exists (11). Notably, the Bitcoin market is particularly attractive to retail investors, who are more sensitive to public information (35). Investor attention can therefore reflect the activities of retail investors in the Bitcoin market capturing new information like the pandemic, making it meaningful to investigate the effect of pandemic attention on Bitcoin returns and volatility.

To answer the issue, the paper employs the daily Google search volume index (GSVI) for the keyword “coronavirus” from January 2020 to February 2022 as an indicator of pandemic attention, following the methods of Smales (11) and Chundakkadan and Nedumparambil (15). The Granger causality test, VAR analysis, and several linear effect analyses are then used to examine the impact of pandemic attention on Bitcoin returns and volatility. This paper further performs the out-of-sample forecasts, which encompass both statistical and economic forecasts, to investigate the prediction of pandemic attention on Bitcoin returns and volatility. It provides a vital step to enhancing our comprehension of returns and volatility in Bitcoin. The findings of this paper can provide investors with a necessary tool to make better-informed decisions and help manage investment risks. Additionally, it can assist policymakers to prevent the Bitcoin market’s risk contagion during market downturns and crises, such as the pandemic.

Similar research to this paper is Bashir and Kumar (36), who discussed the effects of pandemic attention on the returns and volatility of cryptocurrencies. However, they concentrated solely on the spot market and ignored predictions of Bitcoin returns and volatility based on pandemic attention. As a regulated financial derivative, Bitcoin futures holds a dominant position in the price discovery process (37), making it more information-sensitive than the Bitcoin spot market. Moreover, Bitcoin futures make investors easily take a short position, and thus more likely to induce a market crash (38). Importantly, the prediction of Bitcoin returns and volatility has attracted extensive academic interest (39, 40), as returns and volatility are important tools in portfolio optimization, risk management, and financial regulation (41). Furthermore, the risk of a dramatic bubble crash necessitates an accurate prediction of Bitcoin volatility (40, 42). This paper, which uses pandemic attention to forecast Bitcoin returns and volatility, provides a new perspective on previous studies exploring exogenous determinants of Bitcoin (39, 40).

### 1.2. Literature review

This section focuses on two aspects. One is Bitcoin and its determinants, the other is investor attention and its applications.

#### 1.2.1. Bitcoin and its determinants

In recent years, the digital economy has emerged globally (25, 26). Its development has spurred continuous innovation in digital finance, resulting in the development of blockchain technology and cryptocurrencies (43). Based on this technology, Bitcoin, proposed by Nakamoto in 2008, is the first and currently the largest and most representative cryptocurrency (44). By the end of January 2023, Bitcoin’s total market capitalization had reached nearly US $ 450 billion, accounting for approximately 42% of the market share.^2^ Additionally, the Bitcoin futures contract (BTC) was successfully launched by the Chicago Mercantile Exchange (CME) on December 18, 2017, making a new era for Bitcoin trading (44). Numerous studies have highlighted the benefits of using Bitcoin, including improving investors’ risk–return profile (45), offering potential safe-haven characteristics (46), and enabling decentralized payment and transaction facilitation (27). However, some scholars have raised concerns that Bitcoin could be used for illegal trade, terrorism financing, money laundering, and evading capital controls (47), resulting in high financial instability (48) and easy to cause financial shocks (49, 50). Furthermore, Bitcoin’s use of non-renewable energy sources has contributed to carbon emissions (51), leading to resource conflicts and environmental pollution (52). To address these issues and meet the needs of digital economy development, central bank digital currency (CBDC) has emerged, which can promote financial inclusion, innovation, and sustainability (24, 48, 53). Overall, cryptocurrencies such as Bitcoin and CBDC are expected to coexist for an extended period (48).

Although Bitcoin is considered one of the most unpredictable cryptocurrencies (54), scholars continue to investigate the determinants of Bitcoin returns and volatility. Some studies have focused on trading-based or technique indices that are closely related to the features of Bitcoin (39), such as supply and demand conditions (55), mining costs (56), trading volume (57), the daily difference between high and low price (58), and market size (59). Recently, more attention has been paid to exogenous determinants of Bitcoin, including economic policy uncertainty (32), global geopolitical risks (33), investor sentiment (18, 60), macroeconomic conditions (55), and the financial market (34, 55). Notably, a growing body of literature has explored the impact of investor attention on Bitcoin returns and volatility (12, 30, 39, 61–63). For example, Samles (12) suggested that increased investor attention is related to higher returns, more volatility, and larger illiquidity in the Bitcoin market. Li et al. (62) demonstrated that short-term investor attention has a stronger impact on Bitcoin returns in a bearish market due to fear loss aversion. Meanwhile, Figá-Talamanca and Patacca (61) argued that investor attention primarily affects Bitcoin volatility rather than Bitcoin returns. Zhu et al. (30) found that investor attention can effectively improve the prediction performance of Bitcoin returns based on linear prediction models, but does not forecast Bitcoin volatility. Additionally, Wang et al. (39) found that incorporating investor attention can enhance the prediction accuracy of Long Short-Term Memory Networks for Bitcoin returns.

#### 1.2.2. Investor attention and its applications

Traditional asset pricing models assume that information can be immediately incorporated into asset prices through allocating abundant investor attention to the asset (64). However, investor attention is a scarce cognitive resource (65), which is becoming even scarcer in the age of the information explosion. Thus, asset prices only react to new information when investors pay attention to it (66). According to the choice asymmetry theory (67), limited attention to specific events reduces the speed at which new information is incorporated into prices. Increased investor attention can lead to more buying and temporarily higher prices, which then reversed, but do not affect the frequency of selling. However, some studies have demonstrated that increased investor attention to new information can generate more information discovery, leading to temporary price pressure (13). Furthermore, according to Andrei and Hasler (68), carefully (careless) investors can quickly (slowly) incorporate new information into prices, resulting in higher (lower) volatility. In other words, the more attention investors pay, the faster new information flows into the market and the higher the volatility. Hence, investor attention can lead to changes in asset returns and volatility, which has been supported by numerous empirical studies (10–13).

Nowadays, researchers have employed different direct proxies to measure investor attention, such as Google search volume index (69), Twitter post counts (70), and Baidu search volume index (71). This has enabled them to detect the impact of investor attention on asset returns and volatility. Google search volume index is particularly useful for analyzing Bitcoin, as it is an Internet-based cryptocurrency, and investors often collect information through Google (61). Moreover, Google search volume index is highly effective in providing diversified, timely information for investors (30). It has also been extensively used in the literature for explaining and forecasting Bitcoin returns and volatility (30, 39, 69, 72, 73). Notably, the use of Google search volume index as a proxy of investor attention is especially effective in the analysis of specific events (10, 15, 74, 75). As a once-in-a-century catastrophic event, the pandemic has had an immense negative impact on investor expectations (8), which has induced drastic changes in investor attention. These changes could influence investor investment decisions, and ultimately be reflected in financial market performance (7). Moreover, evidence from Google search volume index on the pandemic has demonstrated that it is a good proxy for public attention. Thus, it is reasonable to use the pandemic-related Google search volume index to measure pandemic attention. As a result, a growing number of scholars have investigated the impact of pandemic attention on returns and volatility in stock markets (10, 15, 76), energy markets (77), gold and crude oil markets (17) during the pandemic. Specifically, Wang et al. (10) found that during the pandemic, expected investor attention can explain both realized and fundamental stock market volatility, while unexpected investor attention can only explain realized volatility and is more harmful to the stock market. Chundakkadan and Nedumparambil (15) suggested that investor attention is inversely related to daily returns and generated excess stock market volatility during the pandemic. This is similar to the conclusion of Smales (11). Shear et al. (76) found that the negative impact of investor attention to the COVID-19 pandemic on stock returns is stronger in countries where investors possess higher uncertainty avoidance cultural values.

Based on the available literature, it is likely that pandemic attention has had an impact on the returns and volatility of cryptocurrencies, particularly Bitcoin. However, there have been few studies on the impact of the pandemic attention on Bitcoin. While Bashir and Kumar (36) investigated this issue, they mainly focus on the spot cryptocurrency market and did not consider the predictive power of pandemic attention. This paper therefore aims to investigate the statistical predictive ability of pandemic attention on Bitcoin returns and volatility, and further discusses its economic predictive ability by using the certainty equivalent return (CER).

**Figure 1:**
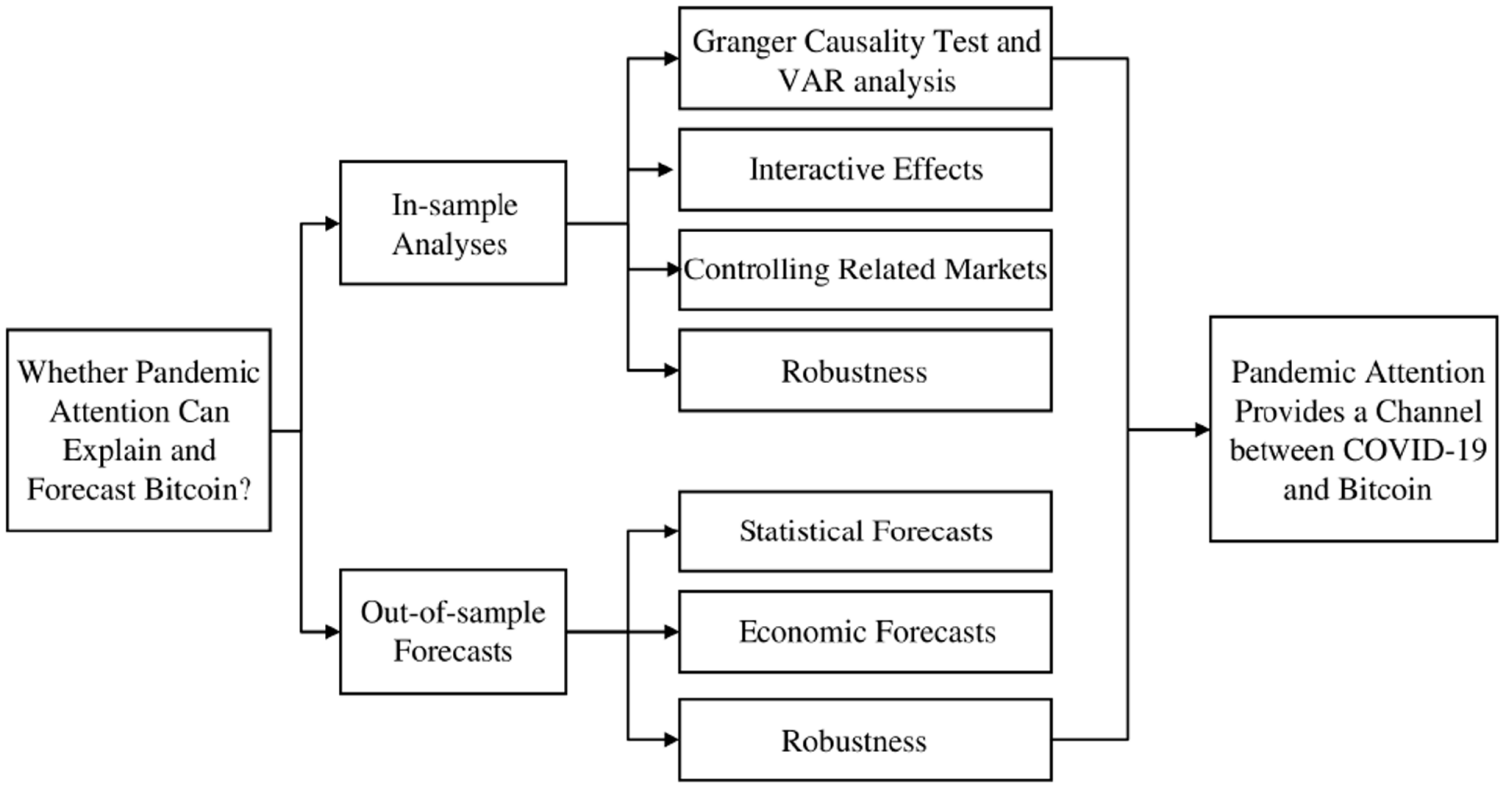
The structure of the paper.

There are two contributions to this paper. First, it enriches the existing literature on the impact of the pandemic on financial markets, specifically the Bitcoin market (18, 31, 78). This paper finds that pandemic attention significantly affects Bitcoin returns and volatility, and that predicting Bitcoin returns and volatility is more accurate when pandemic attention is taken into account. This result adds evidence to the literature on investor attention in extreme events and assists investors in constructing optimal investment portfolios and managing investment risk during crises such as the pandemic. Second, to the best of our knowledge, this paper is the first to examine the impact of pandemic attention on the Bitcoin futures market. In other words, this paper offers a novel perspective to analysis the relationship between the COVID-19 pandemic and Bitcoin, which differs from the analytical perspectives of previous studies, such as the COVID-19 deaths and confirmed cases (23) and the COVID-19 announcements (22). Specifically, the findings suggest that increased pandemic attention leads to a decrease in Bitcoin futures returns and an increase in Bitcoin futures volatility, even after considering the interactive effect and controlling for the influence of other related markets. This paper also enriches the conclusions of Bashir and Kumar (36) by considering the prediction of pandemic attention. It finds that the predictive models that included pandemic attention outperformed the benchmark model on one and longer out-of-sample forecast horizons. Furthermore, the predictive models including pandemic attention have higher average utility and Sharpe ratios than the benchmark model. In summary, pandemic attention plays a crucial and indispensable role in Bitcoin (Figure 1).

The remainder of this paper is organized as follows. Section 2 reports the methodologies and data. Section 3 shows the parameter estimation results of in-sample analyses and its robustness test. Section 4 presents the results of out-of-sample forecasts and its robustness check. Finally, section 5 summarizes the key findings of this paper.

## 2. Methodologies and data

### 2.1. Methods for in-sample analyses

#### 2.1.1. Granger causality test and VAR model

To detect the linear causality relationship between pandemic attention and the returns (volatility) of Bitcoin futures, this paper performs a basic Granger causality test and constructs a VAR model by following previous studies (79–82). Granger causality test can only obtain whether or not a Granger causality relationship exists. To find out when the relationships disappear, or the sign of the relationship, further VAR analysis needs to be conducted (13, 83). The VAR model is able to describe the interactions of the variables by taking each endogenous variable as a function of the lags of all endogenous variables in the economic system (84). Meanwhile, it also can examine the reaction of each variable to the shocks from the remaining variables over time within the impulse response function framework (13). Therefore, it has been widely used to predict the interrelated time-series and analyze the dynamic effects of random perturbations on the system (79–82). In addition, the VAR model’s construction is flexible since it does not need to be based on strict economic theory (84). And more importantly, investor attention can significantly influence asset returns and volatility, and the reverse may also be true (13, 14). Hence, this paper constructs the following equations (1) and (2) to discuss this issue.

(1) $$\begin{array}{c} Bit_{t}=\alpha_{01}+\alpha_{11}Bit_{t-1}+\cdots +\alpha_{n1}Bit_{t-n}\\ +\beta_{11}Att_{t-1}+\cdots +\beta_{n1}Att_{t-n}+\varepsilon_{t} \end{array}$$

(2) $$\begin{array}{c} \;Att_{t}=\alpha_{02}+\alpha_{12}Bit_{t-1}+\cdots +\alpha_{n2}Bit_{t-n}\\ +\beta_{12}Att_{t-1}+\cdots +\beta_{n2}Att_{t-n}+e_{t} \end{array}$$

In the above equations, $Bit_{t}$ represents the returns or volatility of Bitcoin futures at time *t*. $Att_{t}$ is the pandemic attention represented by GSVI. *t-n* is the lag operator for the corresponding variable. In equation (1), the Granger causality test is to statistically test whether the coefficients of ( $\beta_{11},\ldots ,\beta_{n1}$ ) are jointly equal to zero according to a $\chi^{2}$ statistic and its significance level. For equation (2), the null hypothesis of the Granger causality test is that the coefficients of ( $\alpha_{12},\ldots ,\alpha_{n2}$ ) are equal to zero.

#### 2.1.2. Interactive effects

This paper aims to examine whether pandemic attention impacts Bitcoin returns and volatility, so that subsequent research is only based on equation (1). Considering pandemic attention maybe in turn affected by the past performance of Bitcoin returns and volatility, especially dramatically decreased past returns and increased past volatility, the impacts of pandemic attention on Bitcoin returns (volatility) thus to some extent depends on the information (i.e., past returns or volatility) received by the Bitcoin futures market (13, 79–81). For example, when Bitcoin returns and volatility are subject to drastic changes, investors actively look for the influences. Information about the evolutions of the pandemic is largely considered as a possible influence. As a result, the search for information about the pandemic will be intensified, which will generate changes in pandemic attention. Such consideration is supported by previous studies that negative changes in the past performance of specific asset viewed as “bad news” can draw considerable attention (13, 81). According to Vozlyublennaia (13) and Han et al. (79), this paper adds the interaction terms between lagged pandemic attention and Bitcoin returns (volatility) into equation (1) to measure the impact of past returns (volatility) increase or decrease on the effects of pandemic attention. This can also reflect the future reacts of the Bitcoin futures market when a change in its past returns (volatility) value (13). The new equation incorporating the interaction terms is shown in equation (3).

(3) $$\begin{array}{c} Bit_{t}=\alpha_{0}+\alpha_{1}Bit_{t-1}+\cdots +\alpha_{n}Bit_{t-n}+\beta_{1}Att_{t-1}\\ +\cdots +\beta_{n}Att_{t-n}+\delta_{1}Bit_{t-1}*Att_{t-1}\\ +\cdots +\delta_{n}Bit_{t-n}*Att_{t-n}+\varepsilon_{t} \end{array}$$

In equation (3), the coefficients of ( $\delta_{1},\ldots ,\delta_{n}$ ) indicate the increase/decrease of the impact of lagged pandemic attention on Bitcoin returns (volatility) based on unit increases in past Bitcoin returns (volatility). Also, these coefficients can represent the increase/decrease in the impact of past Bitcoin returns (volatility) on current Bitcoin returns (volatility) based on unit increases in past pandemic attention. Moreover, the coefficients of ( $\alpha_{1},\ldots ,\alpha_{n}$ ) measure the effect of past Bitcoin returns (volatility) on current returns (volatility) when pandemic attention remains unchanged. The coefficients of ( $\alpha_{1}+\delta_{1},\ldots ,\alpha_{n}+\delta_{n}$ ) indicate the effect of past Bitcoin returns (volatility) on current returns (volatility), namely Bitcoin returns (volatility) forecasts, under unit increases in past pandemic attention.

#### 2.1.3. Control related markets

According to the existing literature, Bitcoin returns or volatility are affected by stock markets (22, 55), commodity markets (20, 85, 86), and other cryptocurrencies (87). Specifically, the returns of the stock index like the S&P500 index tends to negatively correlate with Bitcoin returns during the pandemic (22), and the stock index is also one of the main factors affecting Bitcoin volatility (55). In addition, there is a negative relationship between crude oil and Bitcoin. When oil prices rise significantly, Bitcoin falls sharply (20). And crude oil and Bitcoin appear a bidirectional Granger-causality relationship under extreme shocks (85). Further, during the pandemic, Bitcoin has been a net receiver of return spillovers from other markets including stock and crude oil, and it has been a net transmitter of volatility spillovers to these markets (86). Finally, cryptocurrencies are becoming increasingly integrated. The increasing interdependence among cryptocurrencies generates a higher level of risk contagion (87). Among these, Bitcoin and Ethereum (ETH) are viewed as the two most popular cryptocurrencies by investors (27), and they are positive correlation (48). Therefore, in order to gain insight into the impact of pandemic attention on the Bitcoin futures market, it is essential to consider the potential influences of other related financial markets by controlling these markets. This paper then constructs a regression model including controlled markets, as shown in equation (4).

(4) $$Bit_{t}=\alpha_{0}+\overset{p}{\underset{i=1}{\sum }}\alpha_{i}Bit_{t-i}+\overset{p}{\underset{i=1}{\sum }}\beta_{i}Att_{t-i}+\overset{p}{\underset{i=1}{\sum }}\gamma_{i}Control_{t-i}+\varepsilon_{t}$$

In equation (4), the lag length *p* is the same as the previous subsections, that is, *p* is set at 4 for Bitcoin returns and 3 for Bitcoin volatility. The control variables include three stock markets (i.e., the NASDAQ, S&P 500, and Dow Jones Industrial Index (DOW) markets), one commodity market (i.e., the WTI crude oil market), and one cryptocurrency market (i.e., the ETH market).

### 2.2. Methods for out-of-sample forecasts

#### 2.2.1. Statistical forecasts

According to Wang et al. (88), good in-sample analysis performance does not imply that the predictive models exist superior out-of-sample performance, as the in-sample analyses may over fitted (82, 88). Conducting out-of-sample forecasts can effectively avoid this problem and provide evidence of prediction performance by following previous studies (79, 88). Moreover, using both in-sample analyses and out-of-sample forecasts can help to establish the robustness of the results (89). This paper thus performs the out-of-sample forecasts to explore whether pandemic attention can predict Bitcoin returns and volatility. In reality, investors are highly interested in the performance of out-of-sample forecasts, as they are more concerned with how well they can do in the future (88). Specifically, this paper divides the total sample of *T* observations for each pandemic attention and Bitcoin returns (volatility) series into an in-sample part containing the first *t* observations and the out-of-sample part containing the remaining *T–t* observations. This paper then uses OLS regressions on the following predictive models (5)–(7) that include pandemic attention to getting out-of-sample forecasts of Bitcoin returns (volatility) on trading day *t + h* (*h* is the forecast horizon, which is set as one that predicts the next trading day) based on the rolling window estimations (88). As for the estimation, the window size is always fixed as the estimation window rolls forward, since the same number of the most distant observations should be discarded after new observations are added to the predictive regression.

(5) $$\begin{array}{c} \overset{}{\hat{Bit_{t+h}}}=\overset{}{\hat{\alpha_{01}}}+\overset{}{\hat{\alpha_{11}}}Bit_{t+h-1}+\ldots +\overset{}{\hat{\alpha_{n1}}}Bit_{t+h-n}\\ +\overset{}{\hat{\beta_{11}}}Att_{t+h-1}+\ldots +\overset{}{\hat{\beta_{n1}}}Att_{t+h-n} \end{array}$$

(6) $$\begin{array}{c} \overset{}{\hat{Bit_{t+h}}}=\overset{}{\hat{\alpha_{0}}}+\overset{}{\hat{\alpha_{1}}}Bit_{t+h-1}+\ldots +\overset{}{\hat{\alpha_{n}}}Bit_{t+h-n}+\overset{}{\hat{\beta_{1}}}Att_{t+h-1}\\ +\ldots +\overset{}{\hat{\beta_{n}}}Att_{t+h-n}+\overset{}{\hat{\delta_{1}}}Bit_{t+h-1}*Att_{t+h-1}\\ +\ldots +\overset{}{\hat{\delta_{n}}}Bit_{t+h-n}*Att_{t+h-n} \end{array}$$

(7) $$\begin{array}{c} \overset{}{\hat{Bit_{t+h}}}=\overset{}{\hat{\alpha_{0}}}+\overset{n}{\underset{i=1}{\sum }}\overset{}{\hat{\alpha_{i}}}Bit_{t+h-i}+\overset{n}{\underset{i=1}{\sum }}\overset{}{\hat{\beta_{i}}}Att_{t+h-i}\\ +\overset{n}{\underset{i=1}{\sum }}\overset{}{\hat{\gamma_{i}}}control_{t+h-i}\; \end{array}$$

To measure the accuracy of different predictive models incorporating pandemic attention, this paper calculates the out-of-sample *R* squared ( $R_{oos}^{2}$ ) and mean squared forecast error adjusted (*MSFE*-adjusted) statistics according to previous studies (80, 82, 90). The $\;R_{oos}^{2}$ represents the proportion of *MSFE* reduction using the predictive models compared to the benchmark. A positive $R_{oos}^{2}$ means that the predictive model is better than the benchmark in forecast performance, while a negative $R_{oos}^{2}$ denotes the opposite. Specifically, the $R_{oos}^{2}\;$ and *MSFE* are expressed as follows,

(8) $$R_{oos}^{2}=1-\frac{\sum_{k=t+h}^{T}{\left(Bit_{k}-\overset{}{\hat{Bit_{k}}}\right)}^{2}}{\sum_{k=t+h}^{T}{\left(Bit_{k}-\bar{Bit_{k}}\right)}^{2}}$$

(9) $$MSFE=\frac{\sum_{k=t+h}^{T}{\left(Bit_{k}-\overset{}{\hat{Bit_{k}}}\right)}^{2}}{T-t-h+1}$$

where *T* represents the observations of the out-of-sample forecast. $Bit_{k}$ denotes the real value of Bitcoin returns (volatility) on day *k*. $\hat{Bit_{k}}$ represents the Bitcoin returns (volatility) forecasted by the predictive models (5)–(7) incorporating pandemic attention. $\bar{Bit_{k}}$ indicates the forecasted returns (volatility) of the benchmark (i.e., historical average).

The *MSFE*-adjusted statistic is proposed by Clark and West (91). It is a one-sided (upper-tail) measurement to test whether the *MSFE* of the benchmark is less than or equal to that of the predictive models. Specifically, the *MSFE*-adjusted statistic is expressed as follows,

(10) $$\begin{array}{c} MSFE\_adjusted=MSFE_{a}-MSFE_{b}\\ +\frac{\sum_{k=t+h}^{T}{\left(\overset{}{\hat{Bit_{k}}}-\bar{Bit_{k}}\right)}^{2}}{T-t-h+1} \end{array}$$

where $MSFE_{a}$ and $MSFE_{b}$ are the *MSFE* statistic of the benchmark and the predictive models incorporating pandemic attention, respectively.

#### 2.2.2. Economic forecasts

Investors, who manage investment portfolios containing Bitcoin, care more about the economic gains of returns (volatility) prediction based on pandemic attention compared with the statistical significance (88). To estimate the economic gains, this paper considers the returns (volatility) prediction as the key determinant of portfolio optimization that maximizes investor utility according to previous studies (79, 88). The effectiveness of returns (volatility) prediction is therefore assessed by observing the portfolio’s performance, namely the utility and Sharpe ratio. This paper then introduces a mean–variance investor who allocates daily weight between Bitcoin futures and risk-free assets by employing returns (volatility) prediction based on the predictive models incorporating pandemic attention, namely equations (5)–(7). Specifically, this paper selects the Secured Overnight Financing Rate (SOFR) as the risk-free asset (92, 93). In addition, the Sharpe ratio is defined as the average portfolio return over the risk-free return divided by the standard deviation of the excess return. The mean–variance investors’ expected utility is shown by equation (11), based on Neely et al. (89) and Wang et al. (88),

(11) $$U_{t}\left(r_{t}\right)=E_{t}\left(w_{t}*r_{t}+r_{t,f}\right)-0.5*\gamma *{\mathrm{var}}_{t}\left(w_{t}*r_{t}+r_{t,f}\right)$$

where $r_{t}$ is the Bitcoin futures returns in excess of the risk-free return denoted by $r_{t,f}$ . $w_{t}$ is the portfolio’s weight of Bitcoin futures, and 𝛾 represents the investors’ risk aversion degree. $E_{t}$ and ${\mathrm{var}}_{t}$ are the return and variance of the portfolio at daily *t*, respectively. At the end of daily *t*, the optimal weight allocated to Bitcoin futures at daily *t + 1* based on maximizing $U_{t}\left(r_{t}\right)$ respect to $w_{t}$ is shown by equation (12),

(12) $$w_{t}^{*}=\frac{1}{\gamma }*\left(\frac{\overset{}{\hat{r_{t+1}}}}{\overset{}{\hat{\sigma_{t+1}^{2}}}}\right)$$

where $\overset{}{\hat{r_{t+1}}}$ and $\overset{}{\hat{\sigma_{t+1}^{2}}}$ are the forecast of 𝑟_𝑡+1_ and its variance, respectively. Observing equation (12), risk aversion degree γ tends to increase means that decrease the weight of Bitcoin futures in the portfolio. According to previous studies (88, 89), the optimal weight is usually between 0 and 1.5. Then the portfolio returns at daily *t + 1* can be written as the equation (13).

(13) $$R_{t+1}=w_{t}^{*}*r_{t+1}+r_{t+1,f}$$

As a popular criterion (88, 89, 94), the certainty equivalent return (CER) is thus introduced to evaluate the performance of investment portfolio containing the Bitcoin futures. And the CER is expressed by the following equation (14).

(14) $$CER_{p}=\overset{}{\hat{\mu_{p}}}-0.5*\gamma *\overset{}{\hat{\sigma_{p}^{2}}}$$

Where $\overset{}{\hat{\mu_{p}}}$ and $\hat{\sigma_{p}^{2}}$ are the mean and variance of the portfolio returns over the period of the out-of-sample forecasts, respectively. The CER can be understood as the risk-free rate of return that investors are willing to accept rather than adopting a risky portfolio (89).

### 2.3. Data

**Figure 2:**
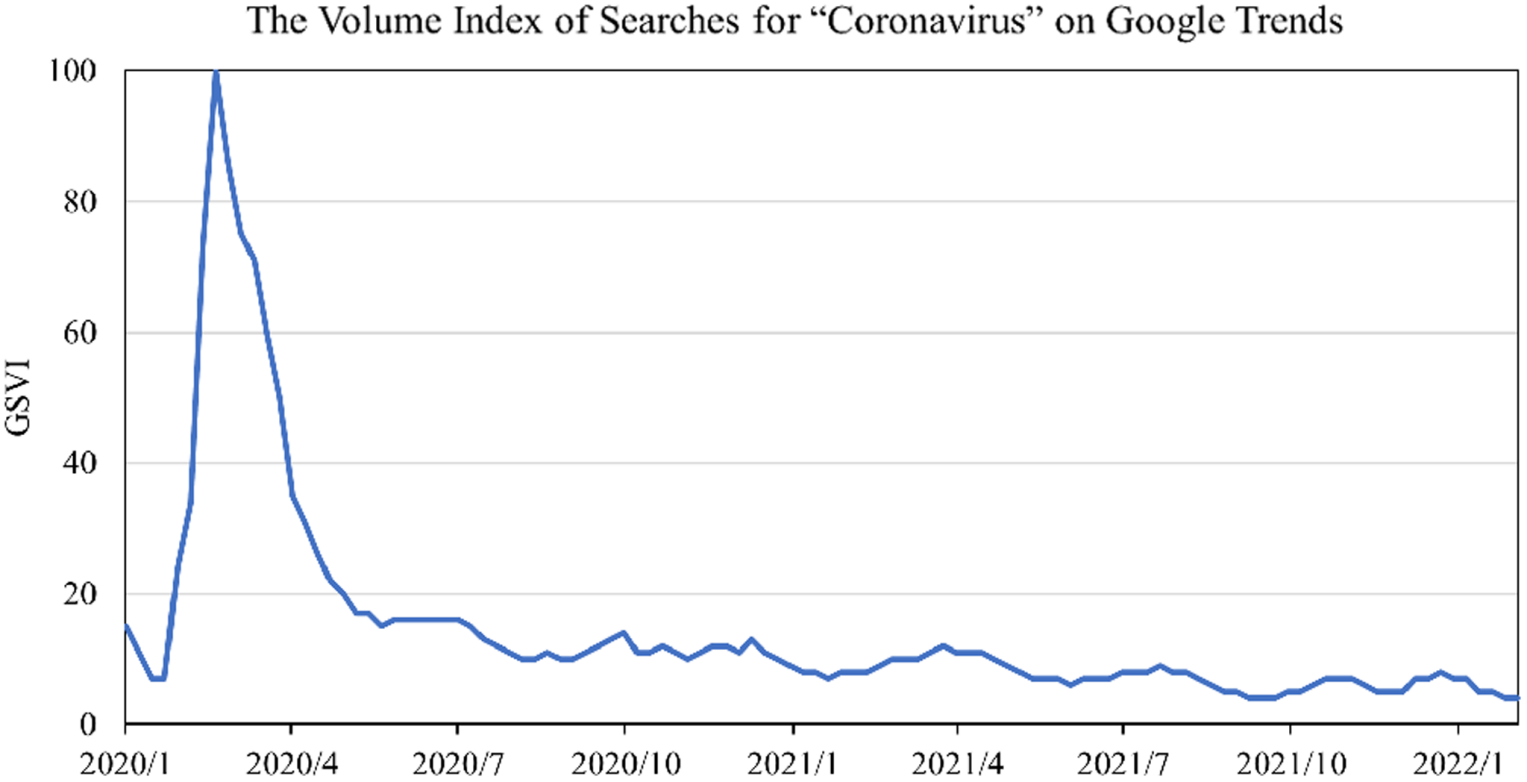
The volume index of searches for “coronavirus” on Google trends.

To measure the pandemic attention, this paper utilizes the Google search volume index (GSVI) on the COVID-19 pandemic following previous studies (10, 17, 77, 95). Specifically, “Worldwide” is set as the search scope, and daily GSVI for “coronavirus” is downloaded from Google Trends^3^ from January 30, 2020 to February 28, 2022. The reason for setting January 30, 2020, as the beginning day of the pandemic, is that the WHO declared the pandemic a public health emergency of international concern on that day,^4^ and thus triggered huge investor attention worldwide. GSVI can provide a time series of search volume indexes ranging from zero to one hundred. Specifically, it is generated by the total number of Google searches on a specific topic over a selected time interval and geographic location (13, 80). The higher the GSVI, the more investors focus on the related search keywords. Figure 2 shows the dynamics of GSVI, which peaked between March 15 and 21, 2020. This may be attributed to COVID-19 being officially declared a pandemic by WHO on March 11, 2020.^5^ After June 2020, the level of GSVI remained relatively low. However, this does not mean that the search volume is low, as the GSVI is scaled by a ratio between 0 and 100. In summary, the GSVI captures well the impact of the pandemic shock on investor psychology or expectations based on massive Internet search data.

As a regulated financial derivative, Bitcoin futures trading is more information-sensitive than spot trading (37). Therefore, this paper utilizes the trading data of Bitcoin futures. The data is collected on a daily basis from Investing.^6^ Following the same approach in previous studies (40, 96), the log-return series of Bitcoin futures prices are calculated as the Bitcoin returns. Specifically, the Bitcoin returns at day *t*, $\;r_{t}$ , is given by:

(15) $$r_{t}=\ln\left(p_{t}\right)-\ln\left(p_{t-1}\right)$$

where $p_{t}$ is the closing price of Bitcoin futures at day *t*. Moreover, the GSVI on the pandemic translates into logarithmic differences for the subsequent empirical processes. This paper further calculates the Bitcoin volatility based on GARCH (*m*, *s*). Specifically, the Bitcoin volatility at day *t*, $\sigma_{t}^{2}$ , is given by:

(16) $$\sigma_{t}^{2}=\alpha_{0}+\overset{m}{\underset{i=1}{\sum }}\alpha_{i}r_{t-i}^{2}+\overset{s}{\underset{i=1}{\sum }}\beta_{i}\sigma_{t-i}^{2}$$

where $r_{t}$ = $\sigma_{t}\varepsilon_{t}$ denotes the Bitcoin returns, $\sigma_{t}^{2}$ is the conditional variance at day *t*, and $\varepsilon_{t}$ is white noise process. The model parameters $\alpha_{0}$ , $\alpha_{i}$ , and $\beta_{i}$ must satisfy stationary conditions. Moreover, *m* and *s* are the model orders to be chosen. This paper selects GARCH (1, 1) to estimate Bitcoin volatility since GARCH (1, 1) has been widely used in existing literature for its superiority over more complex models (96, 97).

Table 1 shows the basic descriptive statistics. It is clear that the GSVI has a higher maximum value and a lower minimum value, which explains the higher standard deviation. The skewness, kurtosis, and Jarque-Bera statistics illustrate that GSVI, Bitcoin returns, and Bitcoin volatility have similar characteristics to the common financial time series, namely “peak thickness and fat tail” and abnormal distribution.

**Table 1:** The descriptive statistics of GSVI on the pandemic and the returns and volatility of Bitcoin futures.

	Mean	Std. dev	Min	Max	Skewness	Kurtosis	Jarque-Bera
GSVI	−0.0026	0.0864	−0.5008	0.6061	1.2605	15.5848	5216.49***
Bitcoin returns	0.0033	0.0418	−0.2349	0.2234	0.0989	6.4642	381.26***
Bitcoin volatility	0.0018	0.0012	0.0005	0.0086	2.2351	9.6291	2024.36***

The descriptive statistics of the GSVI on the pandemic as well as Bitcoin returns and volatility. Jarque-Bera represents the Jarque-Bera statistic, std. dev indicates the standard deviation, and *** represents the significance at the 1% level.

This paper further investigates the prediction ability of GSVI on Bitcoin futures. Hence, the full sample is divided into two parts. The sample period from January 30, 2020 to October 30, 2021 is considered as the in-sample period to analyze the explanatory power of GSVI on Bitcoin futures, while the subsequent period from October 31, 2021 to February 28, 2022 is the out-of-sample forecast.

## 3. The parameter estimation results of in-sample analyses and its robustness test

### 3.1. Granger causality test and VAR results

During the VAR modelling, a key step is to verify the stationary of the selected variables based on the Augmented Dickey-Fuller unit root test (ADF test). The other step is to determine the lag length represented by *n* in equations (1) and (2). Panel A of Table 2 shows that all three of the variables used in this paper are stationary, and thus appropriate for VAR modelling. In addition, according to the LR, FPE, and AIC criteria, panels B and C of Table 2 indicate that the lag lengths of the VAR model on Bitcoin returns and Bitcoin volatility are 4 and 3, respectively.

**Table 2:** Results for the ADF tests and the VAR lag length selections.

Panel A: ADF test results		
	*T*-statistic	Conclusion
GSVI	−23.1412***	Stationary
Bitcoin returns	−22.2099***	Stationary
Bitcoin volatility	−4.5578***	Stationary

Panel B: VAR lag length selection for Bitcoin returns and GSVI				
Lag	LR	FPE	AIC	SC
0	NA	1.42e-05	−5.4846	−5.4706*
1	15.3596	1.41e-05	−5.4963	−5.4543
2	4.0013	1.41e-05	−5.4901	−5.4201
3	9.5951	1.41e-05	−5.4927	−5.394
4	11.3631*	1.40e-05*	−5.4983*	−5.3723
Panel C: VAR lag length selection for Bitcoin volatility and GSVI				
Lag	LR	FPE	AIC	SC
0	NA	1.38e-08	−12.4211	−12.4071
1	1353.7200	1.65e-09	−14.5437	−14.5017*
2	6.9872	1.66e-09	−14.5422	−14.4723
3	10.9270*	1.65e-09*	−14.5470*	−14.4491
4	3.7724	1.66e-09	−14.5405	−14.4145

The results of the ADF stationary tests and VAR lag length selections. *** in Panel A represents the significance at the 1% level. In Panels B and C, LR is a sequentially modified LR test statistic, FPE represents the final prediction error, AIC refers to the Akaike information criterion, and SC denotes the Schwarz information criterion. * in Panels B and C indicates the lag order selected by specified criteria.

Based on the selected lag lengths, this paper performs Granger causality tests and estimates the coefficients in equations (1) and (2). The results are shown in Table 3. First, GSVI is the granger cause of the changes in both the returns and volatility of Bitcoin futures, as the null hypotheses of H1 and H3 are rejected. While Bitcoin returns and volatility are not the granger causes of the GSVI changes. Second, it is evident that GSVI, Bitcoin returns, and volatility are autocorrelated, since some of their lagged terms are statistically significant. Third, GSVI may not have an immediate effect on Bitcoin returns, but it does have a negative impact on Bitcoin returns as $Att_{t-3}$ is significant at the 5% level. This result is similar to that of Chundakkadan and Nedumparambil (15), who argue that investor attention to the pandemic can negatively impact asset returns. This phenomenon may be attributed to increased pessimistic expectations among retail investors due to increased pandemic attention, leading to price pressure (11, 15). Fourth, GSVI has an immediate, positive impact on Bitcoin volatility, as $Att_{t-1}$ is significant at the 1% level. This could be due to anxieties surrounding the economic and financial repercussions of the pandemic, which have brought more pandemic attention and consequently have caused the information to flow faster into the Bitcoin futures market (11). Additionally, trading decisions driven by panic sentiment result in more noise trading and the market’s excess volatility in the short run (15, 98).

**Table 3:** Results for the Granger causality tests and the corresponding VAR estimations.

Panel A: Granger causality tests for Bitcoin returns and GSVI (The VAR lag length is set as 4)	
	$\chi^{2}$ -statistic
Null hypothesis H1: GSVI does not granger cause Bitcoin returns	7.9145*
Null hypothesis H2: Bitcoin returns do not granger cause GSVI	2.0681
Panel B: Granger causality tests for Bitcoin volatility and GSVI (the VAR lag length is set as 3)	
	$\chi^{2}$ -statistic
Null hypothesis H3: GSVI does not granger cause Bitcoin volatility	14.2110***
Null hypothesis H4: Bitcoin volatility does not granger cause GSVI	3.8766

Panel C: VAR estimation results for Bitcoin returns (volatility) and GSVI				
	Bitcoin returns	GSVI	Bitcoin volatility	GSVI
$Bit_{t-1}$	0.1219*** (0.0393)	−0.0400 (0.0826)	1.0262*** (0.0395)	−3.4757 (7.6677)
$Bit_{t-2}$	−0.0126 (0.0394)	−0.0689 (0.0829)	−0.1566*** (0.0562)	16.6921 (10.9151)
$Bit_{t-3}$	−0.0206 (0.0394)	−0.0645 (0.0828)	0.0673* (0.0394)	−14.8302* (7.6495)
$Bit_{t-4}$	0.1249*** (0.0391)	−0.0308 (0.0823)		
$Att_{t-1}$	0.0029 (0.0187)	0.0855** (0.0392)	0.0007*** (0.0002)	0.0888** (0.0394)
$Att_{t-2}$	−0.0264 (0.0187)	−0.0308 (0.0393)	0.0002 (0.0002)	−0.0173 (0.0399)
$Att_{t-3}$	−0.0401** (0.0187)	0.0803** (0.0393)	−0.0001 (0.0002)	0.0667 (0.0398)
$Att_{t-4}$	−0.0131 (0.0188)	−0.0343 (0.0394)		
Constant	0.0036** (0.0017)	−0.0011 (0.0035)	0.0001*** (0.00003)	0.0014 (0.0062)
*R* ^2^	0.0449	0.0179	0.8829	0.0194

Panels A and B in this table report the results from the Granger causality test, and Panel C presents the results of VAR estimation for Bitcoin returns (volatility) and GSVI. The results of the VAR estimation are shown in four columns: the second and fourth columns of Panel C show the results for Bitcoin returns and Bitcoin volatility as dependent variables, respectively. The third and fifth columns show the results of GSVI as the dependent variable. The values in parentheses represent standard errors. *, **, and *** represent the significance levels of 10, 5, and 1%, respectively.

**Figure 3:**
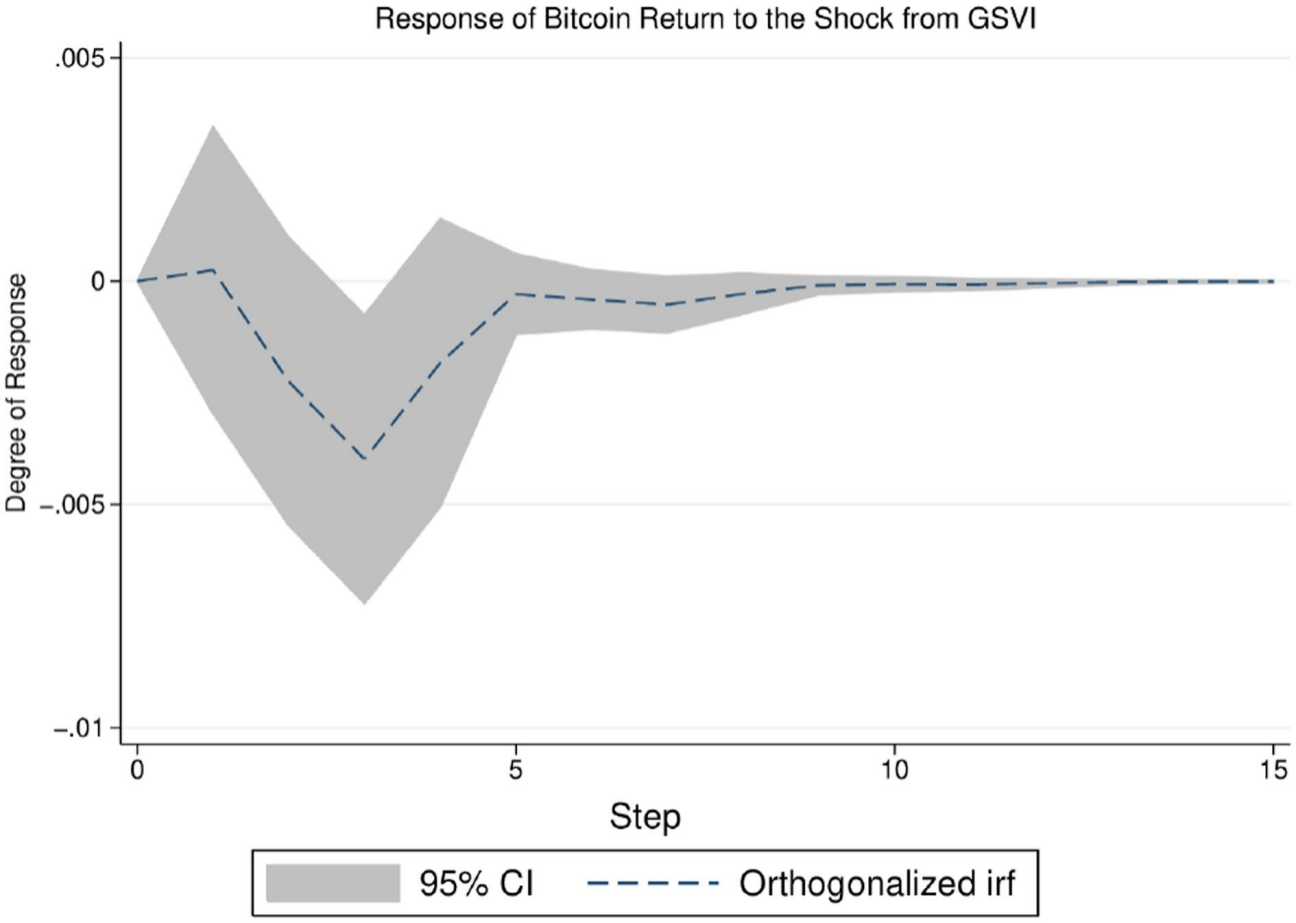
Response of Bitcoin returns to the shock from GSVI.

**Figure 4:**
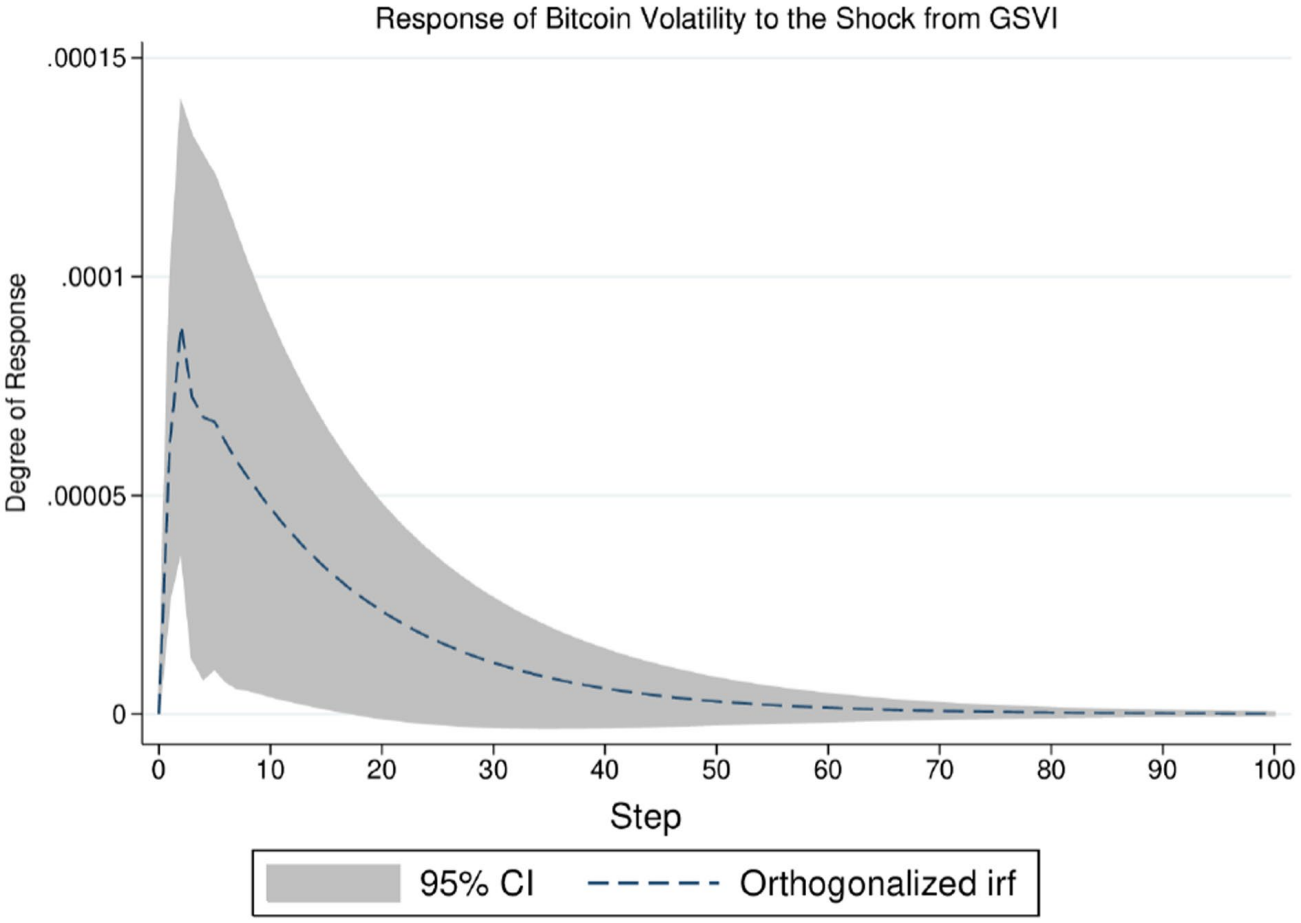
Response of Bitcoin volatility to the shock from GSVI.

Moreover, the VAR analysis offers an impulse response framework to quantify the reaction of one variable to the shock from another. This paper thus applies impulse response analysis to the returns and volatility of Bitcoin futures, aiming to gain insight into the influence of GSVI on the Bitcoin futures market. The detailed results are shown in Figures 3, 4. Figure 3 indicates that the negative impact of GSVI’s unit shock on Bitcoin returns may persist for about 10 days (i.e., more than 1 week), and its peak is reached on the fourth day. Figure 4 presents that the positive effect of GSVI’s unit shock on Bitcoin volatility may last for about 70 days (i.e., 10 weeks), and diminishing rapidly. The relatively short duration of the impact of pandemic attention on Bitcoin return implies that information related to the pandemic could be gradually incorporated into the market, making it more efficient (13). In summary, these findings suggest that the impact of pandemic attention on the returns and volatility of Bitcoin futures merits further analysis.

### 3.2. Interactive effects results

According to the findings of the preceding analysis, GSVI has a significant effect on the returns and volatility of Bitcoin futures. However, GSVI is in turn affected by past Bitcoin returns and volatility performance. In this section, the interactive effects are discussed in detail. According to the lag length identified in the previous subsection, OLS regression is performed for equation (3), and the estimation results are shown in Table 4.

**Table 4:** The estimation results of the interactive effects.

	Bitcoin returns		Bitcoin volatility	
$Bit_{t-1}$	0.1049**	(0.0405)	1.0243***	(0.0399)
$Bit_{t-2}$	−0.0228	(0.0406)	−0.1440**	(0.0568)
$Bit_{t-3}$	−0.0262	(0.0407)	0.0624	(0.0399)
$Bit_{t-4}$	0.1124***	(0.0404)		
$Att_{t-1}$	−0.0011	(0.0191)	0.0005	(0.0004)
$Att_{t-2}$	−0.0299	(0.0191)	−0.0004	(0.0004)
$Att_{t-3}$	−0.0378**	(0.0191)	−0.0005	(0.0004)
$Att_{t-4}$	−0.0150	(0.0192)		
$Bit_{t-1}*Att_{t-1}$	0.5394	(0.3401)	0.1159	(0.2014)
$Bit_{t-2}*Att_{t-2}$	0.2699	(0.3391)	0.3629*	(0.2001)
$Bit_{t-3}*Att_{t-3}$	0.0949	(0.3394)	0.2126	(0.2000)
$Bit_{t-4}*Att_{t-4}$	0.5945*	(0.3400)		
Constant	0.0039**	(0.0017)	0.0001***	(0.00003)
*R* ^2^	0.0549		0.8836	

The estimations results of the interactive effects between lagged GSVI and Bitcoin returns (volatility). The second and fourth columns show the estimation results for Bitcoin returns and Bitcoin volatility as dependent variables, respectively. The values in parentheses in the third and fifth columns represent the standard errors. *, **, and, *** represent the significance levels of 10, 5 and 1%, respectively.

From Table 4, it can be seen that Bitcoin futures are affected by lagged GSVI with changes in past Bitcoin returns (volatility). Specifically, a rise in Bitcoin returns in the preceding 4 days is associated with a notable increase in the impact of GSVI on Bitcoin returns, indicating that more investor attention causes higher Bitcoin returns. Thus, the pandemic has sparked an influx of momentum investors in the Bitcoin futures market who are expecting returns to grow in line with past returns (13). For Bitcoin volatility, an increase in the preceding 2 days leads to a significant increase in the impact of GSVI on Bitcoin volatility, which is similar to the case of Bitcoin returns. Moreover, the significantly positive interaction effect at the fourth lag (the coefficient of $\delta_{4}$ ) corresponds to the positive effect of past Bitcoin returns at the same lag (the coefficient of $\alpha_{4}$ ). Thus, since return forecasting is positively influenced by past GSVI, more GSVI is likely to generate a stronger relation between past and current Bitcoin returns (the coefficient of $\alpha_{4}$ + $\delta_{4}$ becomes greater than $\alpha_{4}$ or $\delta_{4}$ ). However, more GSVI could result in a weaker connection between past and current Bitcoin volatility, since the positive interaction effect at the second lag corresponds to the opposite effect of past Bitcoin volatility at the same lag. This difference implies that as more pandemic attention is given to the Bitcoin futures market, Bitcoin returns are more predictable, whereas Bitcoin volatility is less predictable based on its past performance. To conclude, GSVI is a key factor in the Bitcoin futures market, taking into account interactive effects.

### 3.3. Results after controlling related markets

Table 5 reveals the impact of GSVI and controlled related markets on Bitcoin returns and volatility based on OLS estimations. First, the stock market has a significantly negative effect on Bitcoin returns due to the coefficient of $\gamma_{3}$ is significantly negative. Further, the stock market presents an immediate negative effect on Bitcoin volatility at the first and second lags, and a delayed positive effect at the third lag. These findings support the results of Mariana et al. (22) and Bakas et al. (55). Second, the ETH market has a positive effect on Bitcoin returns at the fourth lag, and an immediate negative effect on Bitcoin volatility at the first lag. This finding enriches the conclusions of Koutmos (87) and Wang et al. (48), who only noted a correlation between Bitcoin and ETH. Third, the WTI crude oil market has had no effect on the Bitcoin market during the pandemic. This result is inconsistent with the findings of previous studies (20, 85, 86), and could be attributed to the introduction of GSVI and other markets that have made crude oil unrelated to Bitcoin. Finally, GSVI has a negative effect on Bitcoin returns and a positive effect on Bitcoin volatility. This implies that the effect of GSVI persists even with the control of other related markets.

**Table 5:** Estimation results after controlling for the related markets.

Panel A: Results for the impact of GSVI and related markets on Bitcoin returns					
	NASDAQ	S&P500	DOW	WTI	ETH
$Bit_{t-1}$	0.1244*** (0.0414)	0.1237*** (0.0412)	0.1227*** (0.0409)	0.1204*** (0.0399)	0.1069** (0.0511)
$Bit_{t-2}$	−0.0186 (0.0421)	−0.0185 (0.0415)	−0.0185 (0.0412)	−0.0120 (0.0403)	−0.0127 (0.0512)
$Bit_{t-3}$	0.0028 (0.0421)	0.0015 (0.0416)	0.0015 (0.0412)	−0.0133 (0.0403)	−0.0119 (0.0511)
$Bit_{t-4}$	0.1144*** (0.0417)	0.1105*** (0.0413)	0.1106*** (0.0410)	0.1203*** (0.0398)	0.0626 (0.0500)
$Att_{t-1}$	0.0036 (0.0194)	0.0060 (0.0194)	0.0069 (0.0193)	0.0053 (0.0190)	0.0063 (0.0189)
$Att_{t-2}$	−0.0277 (0.0192)	−0.0271 (0.0192)	−0.0272 (0.0191)	−0.0257 (0.0189)	−0.0267 (0.0189)
$Att_{t-3}$	−0.0460** (0.0193)	−0.0451** (0.0192)	−0.0448** (0.0191)	−0.0404** (0.0190)	−0.0392** (0.0189)
$Att_{t-4}$	−0.0134 (0.0192)	−0.0113 (0.0192)	−0.0111 (0.0191)	−0.0122 (0.0190)	−0.0113 (0.0189)
$Control_{t-1}$	−0.0125 (0.1073)	0.0135 (0.1141)	0.0297 (0.1074)	0.0056 (0.0162)	0.0223 (0.0392)
$Control_{t-2}$	0.0522 (0.1075)	0.0705 (0.1139)	0.0793 (0.1072)	0.0044 (0.0238)	0.0020 (0.0400)
$Control_{t-3}$	−0.1829* (0.1074)	−0.2008* (0.1140)	−0.2089* (0.1074)	−0.0241 (0.0239)	−0.0123 (0.0400)
$Control_{t-4}$	−0.0024 (0.1071)	0.0554 (0.1146)	0.0625 (0.1081)	0.0175 (0.0163)	0.0754* (0.0392)
Constant	0.0037** (0.0017)	0.0036** (0.0017)	0.0036** (0.0017)	0.0036** (0.0017)	0.0033* (0.0017)
*R* ^2^	0.0496	0.0503	0.0515	0.0478	0.0516
Panel B: Results for the impact of GSVI and related markets on Bitcoin volatility					
	NASDAQ	S&P500	DOW	WTI	ETH
$Bit_{t-1}$	1.0334*** (0.0398)	1.0340*** (0.0398)	1.0311*** (0.0399)	1.0291*** (0.0398)	1.0406*** (0.0401)
$Bit_{t-2}$	−0.1496*** (0.0563)	−0.1468*** (0.0563)	−0.1448** (0.0562)	−0.1623*** (0.0567)	−0.1721*** (0.0574)
$Bit_{t-3}$	0.0512 (0.0394)	0.0465 (0.0393)	0.0465 (0.0392)	0.0704* (0.0397)	0.0712* (0.0399)
$Att_{t-1}$	0.0006*** (0.0002)	0.0006*** (0.0002)	0.0006*** (0.0002)	0.0007*** (0.0002)	0.0007*** (0.0002)
$Att_{t-2}$	0.0001 (0.0002)	0.0001 (0.0002)	0.0001 (0.0002)	0.0002 (0.0002)	0.0002 (0.0002)
$Att_{t-3}$	−0.0001 (0.0002)	−0.0001 (0.0002)	−0.0001 (0.0002)	−0.0001 (0.0002)	−0.0001 (0.0002)
$Control_{t-1}$	−0.0019* (0.0011)	−0.0026** (0.0012)	−0.0025** (0.0011)	0.0002 (0.0002)	−0.0012*** (0.0003)
$Control_{t-2}$	−0.0037*** (0.0011)	−0.0042*** (0.0012)	−0.0044*** (0.0011)	−0.0004 (0.0002)	−0.0003 (0.0003)
$Control_{t-3}$	0.0023** (0.0011)	0.0027** (0.0012)	0.0023** (0.0011)	0.0002 (0.0002)	−0.0001 (0.0003)
Constant	0.0001*** (0.00003)	0.0001*** (0.00003)	0.0001*** (0.00003)	0.0001*** (0.00003)	0.0001 (0.00003)
*R* ^2^	0.8861	0.8869	0.8872	0.8833	0.8854

The impact of GSVI and related markets on Bitcoin returns and volatility. ${\mathrm{Bit}}_{t-i}$ in Panel A refers to Bitcoin returns, while it in Panel B means Bitcoin volatility. The values in parentheses denote the standard errors. *, ** and, *** represent the significance levels of 10, 5, and 1%, respectively.

In summary, pandemic attention is the Grange cause of Bitcoin returns and volatility. Through the analysis of VAR, interactive effects, and control related markets, pandemic attention also has a linear impact on Bitcoin returns and volatility.

### 3.4. Robustness check

To make the main results more rigorous, this paper performs a robustness check by updating the search keywords in Google Trends to generate a new GSVI. The reason for this robustness is that investor attention based on different search keywords may have different or even opposite effects on assets (82).

On February 11, 2020, the WHO announced the novel coronavirus’ official name to be COVID-19.^7^ Therefore, this paper updates the search keyword from “coronavirus” to “COVID-19” and re-performs the related VAR modelling and Granger causality test. Tables 6, 7 present the relevant results, which demonstrate that GSVI on “COVID-19” does granger cause Bitcoin returns and volatility. Moreover, GSVI on “COVID-19” negatively impact Bitcoin returns at the third lag and positively impact Bitcoin volatility at both the first and the second lags. In summary, the pandemic attention does affect Bitcoin returns and volatility, even though investor attention has been updated *via* changing search keywords in Google Trends.

**Table 6:** Bitcoin returns robustness check: updating the search keyword.

Panel A: Stationary test for GSVI on “COVID-19”		
	*T*-statistic	Conclusion
GSVI on “COVID-19”	−17.7000***	Stationary

Panel B: VAR lag length selection for robustness check: the perspective of Bitcoin returns				
Lag	LR	FPE	AIC	SC
0	NA	0.0002	−2.9680	−2.9556
1	40.4165	0.0002	−3.0118	−2.9746
2	69.0581	0.0002	−3.0943	−3.0324
3	36.1455	0.0001	−3.1326	−3.0459*
4	24.3690*	0.0001*	−3.1549*	−3.0435

Panel C: Granger causality tests for robustness check: the perspective of Bitcoin returns	
	$\chi^{2}$ -statistic
H1: GSVI on “COVID-19” does not granger cause the Bitcoin returns	2.9849**
H2: Bitcoin returns do not granger cause the GSVI on “COVID-19”	1.3806

Panel D: VAR estimation results for robustness check: the perspective of Bitcoin returns		
	Bitcoin returns	GSVI
$Bit_{t-1}$	0.1346*** (0.0363)	−0.3475 (0.2559)
$Bit_{t-2}$	−0.0091 (0.0365)	0.2227 (0.2570)
$Bit_{t-3}$	−0.0237 (0.0365)	0.1280 (0.2571)
$Bit_{t-4}$	0.1262*** (0.0361)	−0.4512 (0.2544)
$Att_{t-1}$	−0.0084 (0.0051)	−0.3085*** (0.0362)
$Att_{t-2}$	−0.0074 (0.0053)	−0.3704*** (0.0371)
$Att_{t-3}$	−0.0173*** (0.0051)	−0.2064*** (0.0357)
$Att_{t-4}$	−0.0059 (0.0050)	−0.1001*** (0.0351)
Constant	0.0027* (0.0015)	0.0099 (0.0107)
*R* ^2^	0.0498	0.1638

The robustness check results for Bitcoin returns when updating the search keywords of GSVI. The VAR estimation results are shown in four columns: the second and fourth columns of Panel D are used to present the results for Bitcoin returns and GSVI as dependent variables, respectively, while the third and fifth columns are used to present the corresponding standard errors. * in panel B indicates the lag order selected by specified criteria. ***, **, and * in Panels A, C, and D represent the significance at the 1, 5, and 10% levels, respectively.

**Table 7:** Bitcoin volatility robustness check: updating the search keyword.

Panel A: VAR lag length selection for robustness check: the perspective of Bitcoin volatility				
Lag	LR	FPE	AIC	SC
0	NA	1.56E(−7)	−9.9945	−9.9821
1	1582.6660	1.87E(−8)	−12.1167	−12.0796
2	78.3818	1.70E(−8)	−12.2119	−12.1500
3	29.6821	1.65E(−8)	−12.2414	−12.1547*
4	16.2000*	1.64E(−8)*	−12.2527*	−12.1412

Panel B: Granger causality tests for robustness check: the perspective of Bitcoin volatility	
	$\chi^{2}$ -statistic
H3: GSVI on “COVID-19” does not granger cause Bitcoin volatility	1.9871*
H4: Bitcoin volatility does not granger cause the GSVI on “COVID-19”	0.4836

Panel C: VAR estimation results for robustness check: the perspective of Bitcoin volatility		
	Bitcoin volatility	GSVI
$Bit_{t-1}$	1.0262*** (0.0365)	−24.8202 (24.3716)
$Bit_{t-2}$	−0.1563*** (0.0524)	43.5450 (35.0214)
$Bit_{t-3}$	0.0121 (0.0524)	−8.8930 (35.0463)
$Bit_{t-4}$	0.0556 (0.0365)	−7.2450 (24.3711)
$Att_{t-1}$	0.0001* (0.0001)	−0.3074*** (0.0363)
$Att_{t-2}$	0.0001* (0.0001)	−0.3690*** (0.0372)
$Att_{t-3}$	0.0001 (0.0001)	−0.2050*** (0.0358)
$Att_{t-4}$	0.0001 (0.0001)	−0.0974*** (0.0350)
Constant	0.0001*** (0.00003)	0.0037 (0.0194)
*R* ^2^	0.8805	0.1597

The robustness check results for Bitcoin volatility when updating the search keywords of GSVI. The VAR estimation results are shown in four columns: the second and fourth columns of Panel C are presented the results for Bitcoin returns and GSVI as dependent variables, respectively, while the third and fifth columns are used to present the corresponding standard errors. *In panel A indicates the lag order selected by specified criteria. ***, **, * in Panels B and C represent the significance at the 1, 5 and 10%, levels, respectively.

Up until this point, this paper has concluded that pandemic attention, namely GSVI on “coronavirus” or “COVID-19,” indeed affects the returns and volatility of Bitcoin futures. It is evident that pandemic attention is an interesting and important factor in relation to Bitcoin futures. To be more precise, pandemic attention has a negative impact on Bitcoin returns, yet a positive impact on Bitcoin volatility. This interesting phenomenon may be explained for the following reasons. It may refer to the rise in global death cases and confirmed cases of COVID-19, as well as the emergence and spread of new strains of the virus, which has led to an increase in GSVI on “coronavirus” or “COVID-19.” On the one hand, the increase in GSVI may be seen as a positive sign for investors who view Bitcoin as a safe-haven asset (22, 46). This is because the global public health crisis has caused financial turbulence (7, 76), prompting some safe-haven funds to flow into the Bitcoin market, thus pushing up its price. On the other hand, the rise in GSVI may be viewed as a negative sign for investors who perceive Bitcoin as a universal financial asset. The turmoil in financial markets, caused by the global public health crisis, has caused the price of Bitcoin to fall. Moreover, the Bitcoin market is flooded with retail speculators, who are limited in their abilities to collect and process information (13). This makes it difficult to accurately analyze all the incremental information flooding the market and the proportions of each component (65, 67). As a result, trading activity may become more complex, leading to mixed signals of pandemic attention. As demonstrated by Wen et al. (28), Bitcoin underperformed in comparison to traditional safe-haven assets during the pandemic. This may be attributed to funds flowing out of the Bitcoin market and into other assets with less risk and more liquidity (99), which may explain the negative impact of pandemic attention on Bitcoin returns. As mentioned above, the more complex trading activities and money transfers will likely result in greater Bitcoin volatility.

## 4. The results of out-of-sample forecasts and its robustness test

In this section, the in-sample analysis is extended to the out-of-sample forecasts to explore the potential capabilities of GSVI in forecasting the returns and volatility of Bitcoin futures. Specifically, this paper examines the out-of-sample forecasts from two aspects. The first is to explore whether the predictive models incorporating GSVI statistically outperform commonly used benchmarks in forecasting Bitcoin returns and volatility. Given that previous studies have used the historical average model as the forecasting benchmark for the Bitcoin market (100–102), this paper also adopts this model as the benchmark. The second is to investigate whether the investment portfolios based on the out-of-sample forecasts have higher utility or Sharpe ratios. In keeping consistent with the previous sections, this paper forecasts Bitcoin returns and volatility from October 31, 2021 to February 28, 2022.

### 4.1. Statistical forecasts results

Table 8 presents the outcomes of the out-of-sample forecasts. First, for the prediction of Bitcoin returns, all predictive models have positive $R_{oos}^{2}$ and significant *MSFE*_adjusted statistics. This means that all predictive models have better-forecast performance than the historical average. Analogous results exist in the prediction of Bitcoin volatility, implying that the predictive models incorporating GSVI also improve the predictive power of Bitcoin volatility. Second, for the prediction of Bitcoin returns, the VAR model has the largest $R_{oos}^{2}$ compared to other predictive models. This means that the VAR model relying on lagged GSVI and lagged returns can generate better forecast performance when predicting Bitcoin returns. For the prediction of Bitcoin volatility, the predictive model incorporating GSVI and controlling the ETH market is the best because it has the highest $R_{oos}^{2}$ and significant *MSFE*_adjusted statistic. Third, even for longer-horizon predictions, predictive models incorporating pandemic attention do improve the forecast performance compared to the historical average. Moreover, this forecast performance strengthens the prediction of Bitcoin returns while diminishing the prediction of Bitcoin volatility with increasing forecast horizon.

**Table 8:** Out-of-sample forecasts results for the returns and volatility of Bitcoin futures.

Panel A. Out-of-sample forecasts on Bitcoin returns								*h* = 1		*h* = 2		*h* = 3	
$R_{oos}^{2}$	*MSFE*_adjusted	$R_{oos}^{2}$	*MSFE*_adjusted	$R_{oos}^{2}$	*MSFE*_adjusted		
Interactive	0.0271	1.4671*	0.0246	1.4216*	0.0261	1.4645*
NASDAQ	0.0275	1.6916**	0.0300	1.7629**	0.0355	1.9256**
S&P500	0.0368	2.0093**	0.0376	2.0303**	0.0440	2.2141**
DOW	0.0424	2.0873**	0.0426	2.0916**	0.0489	2.2495**
WTI	0.0466	1.8808**	0.0449	1.8451**	0.0490	1.9300**
ETH	0.0425	1.9031**	0.0419	1.8869**	0.0446	1.9374**

	*h* = 4		*h* = 5	
	$R_{oos}^{2}$	*MSFE*_adjusted	$R_{oos}^{2}$	*MSFE*_adjusted
Interactive	0.0301	1.5184*	0.0339	1.6047*
NASDAQ	0.0355	1.8763**	0.0389	1.9363**
S&P500	0.0451	2.1841**	0.0484	2.2581**
DOW	0.0505	2.2365**	0.0543	2.3424**
WTI	0.0510	1.9516**	0.0549	2.0389**
ETH	0.0485	1.9835**	0.0522	2.0849**

Panel B. Out-of-sample forecasts on Bitcoin volatility								*h* = 1		*h* = 2		*h* = 3	
$R_{oos}^{2}$	*MSFE*_adjusted	$R_{oos}^{2}$	*MSFE*_adjusted	$R_{oos}^{2}$	*MSFE*_adjusted		
VAR	0.8647	11.4368***	0.8646	11.3589***	0.8623	11.2391***
Interactive	0.8648	11.4667***	0.8646	11.3872***	0.8623	11.2675***
NASDAQ	0.8620	11.4437***	0.8622	11.3481***	0.8596	11.2321***
S&P500	0.8614	11.4036***	0.8614	11.3091***	0.8588	11.1970***
DOW	0.8604	11.3863***	0.8605	11.3009***	0.8580	11.1848***
WTI	0.8632	11.4169***	0.8627	11.3381***	0.8603	11.2180***
ETH	0.8669	11.4530***	0.8679	11.3865***	0.8657	11.2691***

	*h* = 4		*h* = 5	
	$R_{oos}^{2}$	*MSFE*_adjusted	$R_{oos}^{2}$	*MSFE*_adjusted
Interactive	0.8605	11.1353***	0.8575	10.9932***
NASDAQ	0.8582	11.1017***	0.8557	10.9506***
S&P500	0.8575	11.0670***	0.8550	10.9164***
DOW	0.8566	11.0547***	0.8540	10.9098***
WTI	0.8583	11.0815***	0.8555	10.9381***
ETH	0.8646	11.1331***	0.8619	10.9933***

The out-of-sample forecast results of the predictive models for Bitcoin returns and volatility. Specifically, VAR refers to the VAR model (including 4 lags for predicting Bitcoin returns, and 3 lags for predicting Bitcoin volatility); Interactive refers to the model that incorporates interactive effects between lagged GSVI and Bitcoin returns and volatility; NASDAQ, S&P500, DOW, WTI, and ETH refer to the models that incorporate GSVI and control related markets. The out-of-sample forecast period is from October 31, 2021 to February 28, 2022. *, **, and *** represent the significance levels of 10, 5 and 1%, respectively.

In summary, the utilization of predictive models with pandemic attention is a feasible way to predict the returns and volatility in the Bitcoin market, as demonstrated by its improved forecast performance. However, it is not guaranteed that a successful predictive model will bring clear economic benefits to investors. Therefore, this paper conducts the following calculation of certainty equivalent return (CER) by constructing portfolios containing bitcoin futures and risk-free assets (88, 89).

### 4.2. Economic forecasts results

This section assumes that risk-averse investors with mean–variance preferences participate in the asset allocation of a portfolio that includes risky Bitcoin assets and risk-free assets. The Secured Overnight Financing Rate (SOFR) is chosen as the risk-free asset (92, 93). Further, this paper assumes that investors make an optimal allocation between SOFR and Bitcoin futures according to the predictive models incorporating pandemic attention. To assure the robustness of economic benefit results, the risk aversion parameter *γ* is set as 3, 6, and 9, respectively. Moreover, to meet practical applications, in this paper, the cost per transaction is added, which is set to 0, 10, and 20 basis points, respectively. Tables 9, 10 display the results of the economic benefits reflected by the utility and the sharp ratio in the allocation exercise of Bitcoin futures based on the out-of-sample forecasts.

**Table 9:** Portfolio performance measures: the perspective of utility.

Panel A: Risk aversion parameter sets to 3					
	Cost	Benchmark	VAR	Interactive	NASDAQ
Utility	0	−0.0040	0.0007	−0.0002	−0.0008
	10	−0.0040	0.0001	−0.0008	−0.0014
	20	−0.0041	−0.0005	−0.0014	−0.0020
		**S&P500**	**DOW**	**WTI**	**ETH**
Utility	0	0.0002	0.0003	−0.0003	0.0010
	10	−0.0003	−0.0002	−0.0008	0.0004
	20	−0.0009	−0.0007	−0.0014	−0.0001

Panel B: Risk aversion parameter set to 6					
	Cost	Benchmark	VAR	Interactive	NASDAQ
Utility	0	−0.0020	0.0004	−0.0000	−0.0001
	10	−0.0020	0.0001	−0.0004	−0.0005
	20	−0.0020	−0.0003	−0.0008	−0.0009
		**S&P500**	**DOW**	**WTI**	**ETH**
Utility	0	0.0003	0.0001	−0.0002	0.0005
	10	−0.0001	−0.0003	−0.0006	0.0001
	20	−0.0005	−0.0006	−0.0010	−0.0002

Panel C: Risk aversion parameter set to 9					
	Cost	Benchmark	VAR	Interactive	NASDAQ
Utility	0	−0.0013	0.0003	0.0000	−0.0000
	10	−0.0013	0.0001	−0.0002	−0.0003
	20	−0.0014	−0.0002	−0.0005	−0.0006
		**S&P500**	**DOW**	**WTI**	**ETH**
Utility	0	0.0002	0.0001	−0.0001	0.0004
	10	−0.0001	−0.0002	−0.0004	0.0001
	20	−0.0003	−0.0004	−0.0006	−0.0001

The portfolio performance measures for investors who use either the benchmark or the predictive models with GSVI to allocate weights in the portfolio that includes Bitcoin futures and SOFR. The utility is the net CER gains when a proportional transaction cost is assumed.

**Table 10:** Portfolio performance measures: the perspective of Sharpe ratio.

Panel A: Risk aversion parameter sets to 3					
	Cost	Benchmark	VAR	Interactive	NASDAQ
Sharpe ratio	0	−0.1084	0.0687	0.0341	0.0209
	10	−0.1085	0.0504	0.0119	0.0031
	20	−0.1086	0.0323	−0.0102	−0.0146
		**S&P500**	**DOW**	**WTI**	**ETH**
Sharpe ratio	0	0.0527	0.0582	0.0388	0.0812
	10	0.0358	0.0422	0.0206	0.0629
	20	0.0190	0.0265	0.0025	0.0439

Panel B: Risk aversion parameter sets to 6					
	Cost	Benchmark	VAR	Interactive	NASDAQ
Sharpe ratio	0	−0.1084	0.0823	0.0540	0.0581
	10	−0.1086	0.0646	0.0329	0.0391
	20	−0.1088	0.0470	0.0119	0.0202
		**S&P500**	**DOW**	**WTI**	**ETH**
Sharpe ratio	0	0.0741	0.0682	0.0603	0.0880
	10	0.0560	0.0512	0.0441	0.0703
	20	0.0380	0.0343	0.0279	0.0526

Panel C: Risk aversion parameter sets to 9					
	Cost	Benchmark	VAR	Interactive	NASDAQ
Sharpe ratio	0	−0.1084	0.0920	0.0669	0.0714
	10	−0.1087	0.0763	0.0484	0.0543
	20	−0.1090	0.0604	0.0299	0.0373
		**S&P500**	**DOW**	**WTI**	**ETH**
Sharpe ratio	0	0.0799	0.0758	0.0729	0.0965
	10	0.0625	0.0596	0.0580	0.0807
	20	0.0451	0.0433	0.0430	0.0648

The portfolio performance measures for investors who use either the benchmark or the predictive models with GSVI to allocate weights in the portfolio that includes Bitcoin futures and SOFR. The Sharpe ratio is the mean portfolio return in excess of the risk-free rate divided by the standard deviation of the excess returns.

Table 9 reports the utility of portfolios which include Bitcoin futures and SOFR. All predictive models that incorporate GSVI perform better than the historical average, as their utilities are higher. Moreover, the VAR model and the predictive model incorporating GSVI and controlling the ETH market have the largest utilities at various transaction costs. Furthermore, the utilities of the two models decrease with an increase in risk aversion, implying that investors’ risk preferences can have an effect on the utility. Finally, the utility decreases as transaction costs increase, but this does little to change the main results above. Table 10 presents the Sharpe ratio of the constructed portfolios. The Sharpe ratio of all predictive models is much higher than the historical average, although the Sharpe ratio decreases as transaction costs increase. Moreover, both the VAR model and the predictive model incorporating GSVI and controlling the ETH market have the highest Sharpe ratios at different transaction costs, which is similar to the utility measure. Overall, using pandemic attention to predict Bitcoin futures could lead to considerable economic benefits through asset allocation exercises. Notably, using the VAR model and the predictive model incorporating GSVI and controlling the ETH market can generate better performance, as the utility and Sharpe ratio of the risk portfolio can be significantly improved compared to the benchmark model.

Overall, this paper concludes that pandemic attention can predict Bitcoin returns (volatility) in both statistical forecasts and economics forecasts. This phenomenon may be attributed to the inefficiency of the Bitcoin futures market (103), making it predictable (18, 30, 39). This paper further provides a new predictor. According to Vozlyublennaia (13), investor attention affects the speed at which information is incorporated into asset prices. Moreover, increased investor attention allows asset prices to react more quickly to new information. Considering investor attention is scarce, investors often display classification learning behavior, thus paying more attention to macro information (67). In reality, information about the pandemic clearly attracts more investor attention, deeply influencing investors’ expectations due to fear of loss aversion (62). Then, investment decisions like short selling in the Bitcoin futures market will be made, thus improving the predictability of Bitcoin returns and volatility. Additionally, this paper finds that the predictive model incorporates pandemic attention and controls the ETH market performs better with higher economic gains. This may be attributed to the increasing integration into the cryptocurrency market (48, 87).

### 4.3. Robustness check

To ensure the robustness of prediction results, this paper further conducts the out-of-sample forecasts including statistical forecasts and economic forecasts by using the updated search keywords “COVID-19.” For the purpose of brevity, this paper only reports the out-of-sample prediction results when the forecast horizon is one and the cost is zero. Related results are presented in Tables 11, 12.

**Table 11:** Robustness checks for out-of-sample forecasts.

	Out-of-sample forecasts on Bitcoin returns		Out-of-sample forecasts on Bitcoin volatility	
	$R_{oos}^{2}$	*MSFE*_adjusted	$R_{oos}^{2}$	*MSFE*_adjusted
VAR	0.0252	1.5417*	0.8700	11.1884***
Interactive	0.0398	1.8376**	0.8724	11.2267***
NASDAQ	−0.0168	0.5777	0.8694	11.1954***
S&P500	−0.0114	0.7641	0.8691	11.1830***
DOW	−0.0028	0.9682	0.8695	11.1779***
WTI	0.0331	1.6418*	0.8677	11.1611***
ETH	0.0352	1.8041**	0.8707	11.1760***

The results of robustness checks on out-of-sample forecasts for Bitcoin returns and volatility based on the forecast horizon being one and updating the search keywords of GSVI. The out-of-sample forecast period is from October 31, 2021 to February 28, 2022. *, **, and *** represent the significance levels of 10, 5 and 1%, respectively.

**Table 12:** Robustness checks for portfolio performance measures.

Panel A: Portfolio performance measures: the perspective of utility					
	Risk aversion	Benchmark	VAR	Interactive	NASDAQ
Utility	3	−0.0039	0.0002	0.0004	−0.0014
	6	−0.0020	8.24E-05	0.0002	−0.0011
	9	−0.0013	5.59E-05	0.0003	−0.0010
	**Risk aversion**	**S&P500**	**DOW**	**WTI**	**ETH**
Utility	3	−0.0018	−0.0015	−7.06E-05	0.0009
	6	−0.0011	−0.0011	−0.0002	0.0004
	9	−0.0011	−0.0011	−0.0002	0.0002

Panel B: Portfolio performance measures: the perspective of Sharpe ratio					
	Risk aversion	Benchmark	VAR	Interactive	NASDAQ
Sharpe ratio	3	−0.1069	0.0500	0.0562	0.0080
	6	−0.1069	0.0621	0.0716	0.0214
	9	−0.1069	0.0729	0.0890	0.0217
	**Risk aversion**	**S&P500**	**DOW**	**WTI**	**ETH**
Sharpe ratio	3	−0.0035	0.0016	0.0391	0.0735
	6	0.0185	0.0167	0.0475	0.0783
	9	0.0091	0.0069	0.0592	0.0843

The results of robustness checks on portfolio performance measures by setting zero cost and updating the search keywords of GSVI.

From Table 11, it is evident that the GSVI on “COVID-19” has significant predictive powers for Bitcoin returns and volatility. It especially holds well for the out-of-sample forecasts on Bitcoin volatility, as all predictive models have positive $R_{oos}^{2}$ and significant *MSFE*_adjusted statistics. These results demonstrate that pandemic attention can predict Bitcoin returns and volatility, even if pandemic attention is altered by changing the Google search keywords. Table 12 further confirms that all predictive models with GSVI perform better than the historical average, as the utilities and Sharpe ratios of the predictive models are relatively higher, particularly for the predictive model with interactive by GSVI and Bitcoin returns, as well as the predictive model incorporating GSVI and controlling the ETH market. In summary, using pandemic attention to predict Bitcoin futures is statistically significant and has the potential to generate considerable economic benefits through asset allocation exercises, even when changed by updating the Google search keyword.

## 5. Conclusion

This paper aims to examine whether pandemic attention can affect and predict Bitcoin returns and volatility. To investigate this, the keyword “coronavirus” from Google Trends is used as an indicator of pandemic attention, and the Granger causality test, the VAR analysis, and several linear effects analyses are conducted. The results are as follows. Firstly, pandemic attention does granger cause Bitcoin returns and volatility. Secondly, pandemic attention negatively impacts Bitcoin returns and positively affects Bitcoin volatility. And, whilst the negative impact on Bitcoin returns has a shorter duration, the positive impact on Bitcoin volatility is sustained for a longer period. Thirdly, this paper further performs the out-of-sample forecasts based on the good explanatory power of pandemic attention, and finds that the predictive models including pandemic attention outperform the benchmark model. Moreover, the prediction performance of Bitcoin returns improves, while the prediction performance of Bitcoin volatility decreases as the forecast horizon expands. Fourth, the predictive models seem to have good economic benefits, especially the VAR model and the model that incorporate pandemic attention and control of the ETH market, when constructing portfolios that include Bitcoin futures and risk-free assets. In summary, using pandemic attention can help to explain and predict Bitcoin returns and volatility.

The policy implications of this paper are threefold. First, for theoretical analysis, this paper provides a novel perspective, namely pandemic attention, to analyze and predict Bitcoin returns and volatility. This provides a channel between the global public health crisis and the Bitcoin market. In addition, this paper provides empirical evidence that the global public health crises have a significant influence on the financial markets, particularly the Bitcoin market. Moreover, scholars can use the pandemic attention index or other related indexes for their analysis and decision-making. Second, for professional Bitcoin traders, the pandemic attention proposed in this paper can be considered an effective tool for monitoring the Bitcoin market. It can help to predict Bitcoin during market downturns and crises (such as the COVID-19 pandemic), and devise a trading strategy to seek profits. Meanwhile, the results of this paper suggest that retail investors should exercise caution when buying and selling in Bitcoin, and adjust the weight of their investments in Bitcoin when pandemic attention is high. Thirdly, for regulators, pandemic attention can be included in market monitoring indicators. In terms of market turmoil, regulators can propose appropriate policies for risk pre-warning by monitoring pandemic attention, thus aiding investors to reduce or even avoid investment losses, and prevent the risk contagion in the cryptocurrency market. Furthermore, regulators could rely on investigations of interactions between pandemic attention and the Bitcoin futures market to identify levels of irrational exuberance in the market. Finally, although the sensitivity of global investors and authorities to the pandemic has gradually decreased, this paper can provide guidance for further research and trading on the Bitcoin market based on investor attention caused by public emergencies.

There may be certain limitations in this paper. First, the construction of pandemic attention may be relatively simple. For instance, pandemic attention can be composed of more search keywords and its construction method can be further improved. Moreover, pandemic attention is constructed globally rather than distinguishing different countries (or regions), making it difficult to investigate the variances in the impact of the pandemic on investors from different geographic categories (15). Thus, further research can include more related keywords (104) and introduce the construction methods of Principal Component Analysis (18) and Partial Least Squares regression (80) as well as construct pandemic attention indexes of different regions. Second, pandemic attention does not make a distinction between its positive and negative nature. In reality, pandemic attention holds different information for various investors. As a consequence, further research can differentiate the various impacts of the positive and negative nature of pandemic attention on Bitcoin, and further assess the hypothesis of Barber and Odean (67) and Vozlyublennaia (13) to determine which one is more suitable to explain the Bitcoin market during the pandemic. Third, pandemic attention does not take into account intraday high-frequency information. For high-frequency traders, high-frequency pandemic attention makes more sense. Thus, subsequent research can follow Meshcheryakov and Winters (105) to generate this high-frequency index, and then performs intraday Bitcoin prediction. Fourth, due to the intricate relationship between pandemic attention and Bitcoin returns and volatility, using simple linear models is not sufficient. The development of artificial intelligence has partially overcome the limitations of linear models by introducing machine learning approaches. As a result, subsequent research can consider combining machine learning methods and pandemic attention like Wang et al. (39) to improve the prediction accuracy of Bitcoin returns and volatility. Finally, given the importance of pandemic attention in the financial markets, future research could consider the important role of pandemic attention when investigating risk spillovers across the cryptocurrency markets. This could be a line of research worth considering.

## Data availability statement

Publicly available datasets were analyzed in this study. This data can be found here: The datasets (Pandemic Attention) for this study can be found in the (http://www.Google.com/trends). And the datasets (Trading Data of Bitcoin Futures) for this study can be found in the (https://cn.investing.com/).

## Author contributions

JW: data analysis, methodology, software, formal analysis, writing-original draft preparation, and writing-review and editing. YW: conceptualization, project administration, writing-original draft preparation, and writing-review and editing. PZ: data curation, methodology, software, and writing-original draft preparation, writing-review and editing. All authors contributed to the article and approved the submitted version.

## Funding

This research is supported by the Research Foundation for Youth Scholars of Beijing Technology and Business University under no. QNJJ2020-36.

## Conflict of interest

The authors declare that the research was conducted in the absence of any commercial or financial relationships that could be construed as a potential conflict of interest.

## Publisher’s note

All claims expressed in this article are solely those of the authors and do not necessarily represent those of their affiliated organizations, or those of the publisher, the editors and the reviewers. Any product that may be evaluated in this article, or claim that may be made by its manufacturer, is not guaranteed or endorsed by the publisher.

## Footnotes

^1^ https://covid19.who.int/

^2^ https://coinmarketcap.com/charts/

^3^ http://www.Google.com/trends

^4^ https://www.who.int/director-general/speeches/detail/

^5^ https://www.who.int/emergencies/diseases/novel-coronavirus-2019/interactive-timeline

^6^ https://cn.investing.com/

^7^ https://www.who.int/director-general/speeches/detail/
